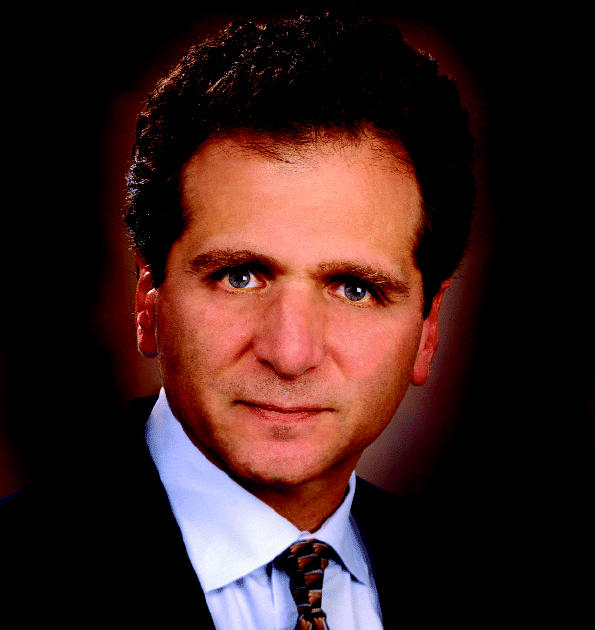# A Vision for the Future

**Published:** 2005-04

**Authors:** David A. Schwartz

**Affiliations:** Director, NIEHS, E-mail: schwartz@niehs.nih.gov

I am honored to step into the leadership of the NIEHS. As the institute’s fourth director, I will strive to maintain the tradition of strong, insightful, and forward-thinking leadership of my predecessors Paul Kotin (1969–1971), David Rall (1971–1990), and Kenneth Olden (1991–2005). While the challenges and research opportunities in the future will be decidedly different from those faced by the institute’s previous leaders, the same principles of dedication to outstanding high-impact science, community participation, accountability, and transparency in the decision-making process will guide my vision for environmental health sciences and tenure as the NIEHS director.

As director, I will avail myself of many ways to convey information about NIEHS research, programs, and perspectives, including frequent communications in *EHP*. With the strong appreciation that the best solutions are often formulated after considering divergent perspectives, I also welcome your thoughts and encourage dialogue with me and my office. This column in *EHP* represents only one of the many ways I plan to interact with our broader community of environmental health scientists, policy makers, other government agencies, interested organizations, and the American public.

By way of introduction, I grew up on Long Island. I was an undergraduate at the University of Rochester in New York, and went to medical school at the University of California–San Diego. I extended my medical, fellowship, and research training at Boston City Hospital, the Harvard School of Public Heath, and the University of Washington, and served as a faculty member at the University of Iowa and Duke University. My wife, Louise Sparks, is a physician who is currently running a local volunteer foster care program. Our three children—Kiera, Sam, and Tziporah—are the pride of our lives (I could talk forever about our children) but continually challenge our reserves.

The very question that patients and families frequently ask me—why does one person develop an illness when challenged with an environmental agent while another person remains healthy?—represents the focus of my research and has led me to the NIEHS.

As a physician–scientist, I have received formal training in internal medicine, pulmonary medicine, environmental health sciences, and genetics. My mentors have included Abraham Braude, Ruth Heifetz, Linda Rosenstock, Gil Omenn, Joan Clark, Gary Hunninghake, Jim Merchant, Jeff Murray, and Frank Abboud—yes, it took a village to train me. I have focused my research on the biology and genetics of environmental lung disease and host defense. These research efforts have provided new insights into the pathophysiology, biology, and genetics of pulmonary fibrosis, endotoxin-induced airway disease, and innate immunity.

While at Duke, I played a principle role in developing three interdisciplinary programs: the Environmental Health Sciences Research Center, the Center for Environmental Genomics, and a program studying the pathogenesis and genetics of environmental asthma. In many respects, my own research reflects the opportunities and challenges created by the progressive leadership at the NIEHS—Ken Olden, Sam Wilson, Anne Sassaman, and Lutz Birnbaumer. I am proud of my background and will draw on my experience as a physician–scientist to lead the NIEHS.

In fact, while directing the NIEHS, I plan to continue to function as a physician–scientist. The reasons for this are simple and straightforward—I enjoy patient care and research, I see these activities as part of my identity, and I believe that the NIEHS will benefit from my perspective as an active physician–scientist. My research has been driven and inspired by taking care of patients with unexplainable illnesses. The very question that patients and families frequently ask me—why does one person develop an illness when challenged with an environmental agent while another person remains healthy?—represents the focus of my research and has led me to the NIEHS, where it is important that we recognize and remember that the ultimate goal of the work that we do is to prevent disease and improve human health.

As director of the NIEHS, I have a unique opportunity to help shape the future of the environmental health sciences. I fully embrace the challenges and opportunities that lie ahead, and I hope to further vitalize the NIEHS by enfolding new and exciting areas of growth and development into this field.

In my role as director, I will strive to:

- provide scientific leadership for the NIEHS;
- create opportunities to understand how human health is affected by environmental exposures and how this knowledge can be used to reduce morbidity, improve quality of life, and extend longevity;
- create a forum for open and ongoing communication between the NIEHS and both the public and scientific communities to identify areas for programmatic development, to fully evaluate a problem or opportunity, and to enhance public and scientific awareness of the importance of environmental exposures to human health;
- develop complementary research and training programs in the extramural and intramural research communities, and, when appropriate, coordinate these activities with programmatic efforts in the National Toxicology Program;
- work with the other institutes of the NIH, the Centers for Disease Control and Prevention, the Environmental Protection Agency, and other relevant scientific agencies to coordinate scientific and training programs;
- foster the development of programmatic, interdisciplinary research in the environmental sciences, environmental public health, and environmental medicine;
- support the development of research careers in the environmental sciences, environmental public health, and environmental medicine; and
- serve as a spokesperson for the environmental health sciences.

I am very excited about my role in the future of the environmental health sciences and am fully committed to serving as the director of the NIEHS. I have decided to take on this responsibility because of my long-standing commitment to this scientific field, and my firm belief in our collective responsibility to advance society. I look forward to the work ahead.

## Figures and Tables

**Figure:**